# Supplementary material for: Complexome profiling of the Chlamydomonas psb28 mutant reveals TEF5 as an early PSII assembly factor
Source: Plant Cell. 2025 Mar 17;37(6):koaf055. doi: 10.1093/plcell/koaf055 (PMC12164590; doi:10.1093/plcell/koaf055)
Supplement: koaf055_Supplementary_Data [file koaf055_supplementary_data.zip › psb28_interactive.zip!/plots/intensity/qualityPlots/peptides/HBD1_Cre12.g495850.t1.2.html]

### Cre12.g495850.t1.2

Trivial name: HBD1  
SeqCoverage [median]: 50.80  
Mass: 27.82 kDa  
  
 

| Sequence | MissedCleavage | Mass [Da] | wtIntensity | muIntensity | Score |
| --- | --- | --- | --- | --- | --- |
| AALITGSTQGIGLGVLK | 0 | 1597.94 | 6011.31 | 9554.10 | 270.45 |
| AALPHMLQQGWGR | 0 | 1463.75 | 4063.36 | 8867.86 | 191.52 |
| DVMLADQPTK | 0 | 1116.55 | 19661.72 | 30807.46 | 200.60 |
| GIPVEAVIR | 0 | 952.57 | 17890.20 | 59980.60 | 119.29 |
| GIPVEAVIRDVMLADQPTK | 1 | 2051.11 | 0.00 | 6323.27 | 47.81 |
| HGVAGFTK | 0 | 815.43 | 8823.77 | 22303.66 | 167.25 |
| IRGIPVEAVIR | 1 | 1221.76 | 3380.48 | 15602.32 | 99.75 |
| NQIADTAR | 0 | 887.45 | 22328.29 | 22438.11 | 142.79 |
| QTADSFGR | 0 | 880.40 | 5477.60 | 7277.50 | 147.04 |
| SAGGILR | 0 | 672.39 | 4140.73 | 6343.86 | 132.96 |
| SAGGILRGK | 1 | 857.51 | 329.62 | 912.63 | 101.64 |
| SAYNAAK | 0 | 723.36 | 4544.32 | 7019.18 | 143.02 |
| TVALEVATK | 0 | 930.54 | 32384.13 | 45624.80 | 118.50 |
| VAYSEADLLKPAAIR | 1 | 1615.89 | 5398.15 | 26720.65 | 83.05 |
| VAYSEADLLKPAAIRDMVK | 2 | 2089.12 | 1760.96 | 41184.08 | 96.67 |
| VVNTGSMHALVASPFK | 0 | 1656.87 | 24594.95 | 58267.46 | 124.25 |
|  |  |  | 160789.6 | 369227.5 |  |
